# Optimization of Preload in Severe Sepsis and Septic Shock

**DOI:** 10.1155/2012/761051

**Published:** 2012-08-07

**Authors:** Adil Shujaat, Abubakr A. Bajwa

**Affiliations:** Division of Pulmonary and Critical Care Medicine, University of Florida College of Medicine-Jacksonville, 655 West 8th Street, Suite 7-088, Jacksonville, FL 32209, USA

## Abstract

In sepsis both under- and overresuscitation are associated with increased morbidity and mortality. Moreover, sepsis can be complicated by myocardial dysfunction, and only half of the critically ill patients exhibit preload responsiveness. It is of paramount importance to accurately, safely, and rapidly determine and optimize preload during resuscitation. Traditional methods of determining preload based on measurement of pressure in a heart chamber or volume of a heart chamber (“static” parameters) are inaccurate and should be abandoned in favor of determining preload responsiveness by using one of the “dynamic parameters” based on respiratory variation in the venous or arterial circulation or based on change in stroke volume in response to an endogenous or exogenous volume challenge. The recent development and validation of a number of noninvasive technologies now allow us to optimize preload in an accurate, safe, rapid and, cost-effective manner.

## 1. Introduction

It is well known that underresuscitation is associated with increased morbidity and mortality in septic shock, and volume resuscitation to optimize preload to improve cardiac output (CO) and blood pressure (BP) is of paramount importance when there is hypovolemia caused by vasodilatation, transudation of fluid into the extravascular compartment, increased insensible fluid loss, and decreased oral intake, in sepsis. On the other hand overzealous resuscitation can also lead to increased morbidity and mortality [1–5]. Moreover, myocardial depression plays a significant role in the pathophysiology of shock in up to 60% of septic patients and can develop at an early stage [6]. Lastly, only about half of the critically ill patients exhibit preload responsiveness [7]—defined as the ability of the heart to increase its stroke volume (SV) in response to an increase in preload. Hence, it is vital that resuscitation in sepsis be guided by accurate assessment and monitoring of hemodynamic status of individual patients.

Traditional methods of determining the adequacy of volume resuscitation have relied on one or another measure of preload, that is, central venous pressure (CVP), pulmonary artery wedge pressure (PAWP), right ventricular end-diastolic volume index (RVEDVI), left ventricular end-diastolic area index (LVEDVAI), and global end-diastolic volume (GEDV)—also known as static parameters of volume status. However, none of these is accurate in predicting preload responsiveness [7–9]. Both pressure and volume measures of preload are affected by multiple factors other than the volume of blood, for example, vascular tone, intrathoracic pressure, and ventricular compliance. Moreover, the Frank-Starling relationship depends upon preload as well as ventricular function. Therefore, it is physiologically impossible to accurately predict preload responsiveness by assessing preload alone. Over the last two decades there has been a paradigm shift in the approach to predicting hypovolemia from measuring preload to actually determining preload responsiveness. Preload responsiveness can be determined by performing a volume challenge maneuver or by making use of the respiratory variation in the venous or arterial circulation—also known as dynamic parameters of volume status. 

The volume challenge maneuver comprises a volume challenge and measurement of an end-point, that is, CVP, BP, SV, CO, heart rate (HR), or urine output. The volume challenge can be in the form of an actual administration of intravenous fluid (exogenous and irreversible volume challenge) or a virtual volume challenge where an endogenous volume of blood is displaced from the legs during passive leg raising (PLR) maneuver (endogenous and reversible volume challenge). It is important to realize that SV (or CO), or its surrogate, for example, pulse pressure (PP) or arterial blood flow velocity, is the preferred end-point because a preload responsive heart may not be recognized otherwise. The SV (or CO) can be measured by invasive or noninvasive methods. 

Use of respiratory variation in the central venous circulation to predict preload responsiveness comprises measurement of CVP or ultrasonographic measurement of the diameter of either vena cava whereas use of respiratory variation in the arterial circulation to predict preload responsiveness comprises measurement of PP, SV, pulse oximeter plethysmographic (POP) waveform amplitude, or arterial blood flow velocity.

Dynamic parameters of volume status outperform the static ones in predicting preload responsiveness and should be used to optimize preload in severe sepsis and septic shock (Table 1) [7, 10].

## 2. Dynamic Parameters Used to Predict Preload Responsiveness

1. Respiratory variation in,
  - central venous pressure (CVP),
  - vena cava diameter,
    - inferior vena cava (IVC),
    - superior vena cava (SVC),
  - arterial blood pressure waveform-derived variables,
    - pulse pressure variation (PPV),
    - stroke volume variation (SVV),
  - pulse oximeter plethysmographic (POP) waveform amplitude,
  - arterial blood flow velocity,
    - aortic,
    - brachial artery,
2. passive leg raising (PLR) maneuver,
3. actual fluid challenge,

## 3. Respiratory Variation in CVP

**Figure 1:**
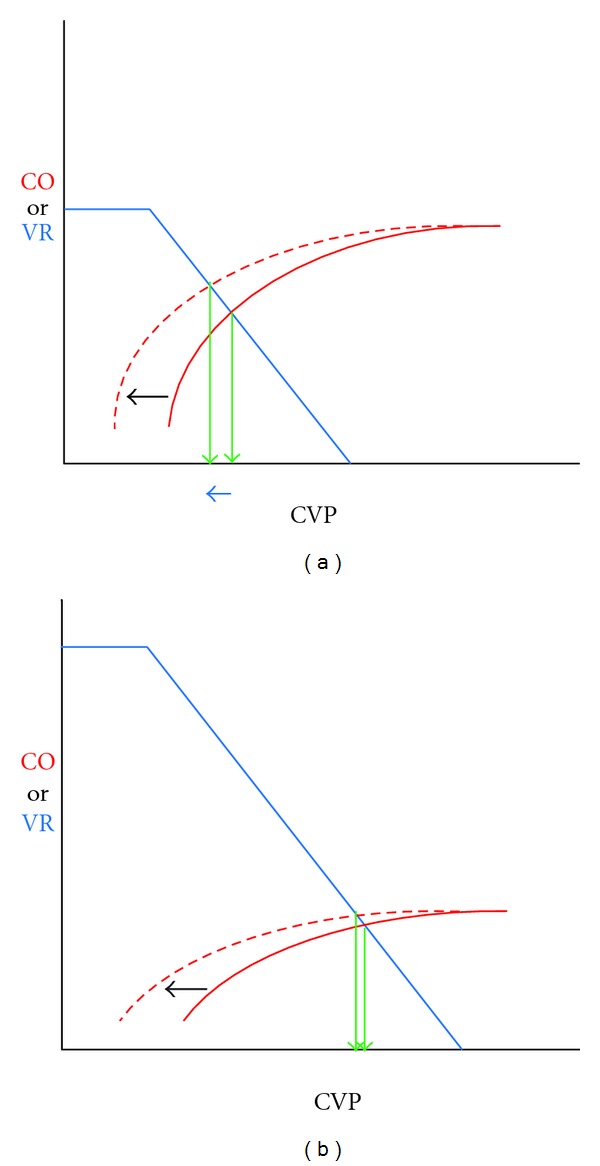
During spontaneous breathing the cardiac function curve (solid red curve) is shifted to the left (dashed red curve). When the heart is functioning on the ascending part of the cardiac function curve. CVP falls (blue arrow) and CO rises ((a), on left). However, when the heart function is depressed or the circulation is volume loaded ((b), on right), CVP and CO remain unchanged. *CVP*: central venous pressure, CO: cardiac output.

Although no single value of CVP can accurately predict preload responsiveness, respiratory changes in CVP can do so. During spontaneous breathing the respiratory changes in pleural pressure can cause cyclic changes in CVP when the right ventricle (RV) is preload responsive than when it is not. An inspiratory fall in CVP indicates that the heart is functioning on the ascending part of the Frank-Starling curve and may or may not respond to volume depending upon how close CVP is to the plateau, whereas lack of an inspiratory fall indicates that the heart is functioning on the flat part of the Frank-Starling curve and will not respond to volume infusion. Therefore, this test is most useful in the negative (see Figure 1).

Magder et al. [11] showed that the lack of an inspiratory fall in CVP of ≥1 mmHg predicted lack of preload responsiveness in spontaneously breathing patients including patients triggering breaths on mechanical ventilation. The converse was less predictive; that is, patients who had an inspiratory fall in CVP of >1 mmHg did not always have an increase in CO. In that study volume loading increased the cardiac output in only 1 of 14 patients who did not have an inspiratory fall in CVP whereas it increased the cardiac output in 16 out of 19 patients who had an inspiratory fall in CVP. 

Use of respiratory variation in CVP to predict preload responsiveness requires that the inspiratory effort be significant enough to cause a 2 mmHg drop in PAWP, and therefore in the absence of a pulmonary artery catheter (PAC) to confirm such a significant respiratory effort the technique becomes subjective and dependent on observing the patient. Moreover, in a patient who is using expiratory abdominal muscles the release of abdominal muscle contraction may be confused for an inspiratory fall in CVP [12].

## 4. Respiratory Variation in Diameter of Either Vena Cava

During mechanical ventilation the cyclic effect of positive airway pressure can cause respiratory variation in the diameter of both the SVC and the IVC. This cyclic effect depends upon the transmural pressure of the vessel which is determined by the intravascular pressure—that, in turn, depends on the circulating blood volume and RV function—and by the surrounding pressure, that is, pleural pressure for SVC and abdominal pressure for IVC since the intrathoracic part of the latter vessel is virtual. The SVC diameter is minimal during inspiration and maximal during expiration. On the other hand, since only a minor proportion of positive airway pressure is transmitted to the abdomen, the IVC diameter is maximal during inspiration and minimal during expiration [13].

In two separate studies [14, 15] of septic patients who were on mechanical ventilation, deeply sedated, receiving a tidal volume of ≥8 mL/kg predicted body weight (PBW) and in normal sinus rhythm, respiratory changes in the IVC measured by M-mode ultrasonography (US) were found to be highly accurate in predicting preload responsiveness—defined as an increase in cardiac index (CI) of ≥15% measured by transthoracic echocardiography (TTE). Barbier et al. [14] showed that in the 23 patients in their study an IVC distensibility index of >18% predicted preload responsiveness with a sensitivity of 90% and specificity of 90%. Feissel et al. [15] showed that in the 39 patients in their study an IVC distensibility index of ≥12% predicted preload responsiveness with an NPV of 93% and PPV of 92%. The threshold value of IVC distensibility index is different because the index was calculated differently—Barbier et al. [14] calculated the index as the difference in the maximum diameter of the IVC at end-inspiration and the minimum diameter at end-expiration divided by its minimum diameter at end-expiration and expressed as a percentage whereas Feissel et al. [15] calculated the index as the difference in the diameter of the IVC at end-inspiration and at end-expiration divided by the mean of the two diameters and expressed as a percentage. 

In a study of septic patients who were on mechanical ventilation, deeply sedated, receiving a tidal volume of ≥8 mL/kg PBW and in normal sinus rhythm, respiratory changes in the SVC measured by transesophageal echocardiography (TEE) were highly accurate in predicting preload responsiveness—defined as an increase in CI of ≥11%. Vieillard-Baron et al. [16] showed that in the 66 patients in their study an SVC collapsibility index of >36% predicted preload responsiveness with a sensitivity of 90% and specificity of 100%. SVC collapsibility index was calculated as the difference in the maximum diameter of the SVC at end-expiration and minimum diameter at end-inspiration divided by its maximum diameter at end-expiration and expressed as a percentage.

SVC is more reliable because it is surrounded by pleural rather than abdominal pressure. However, it can only be adequately visualized by TEE. Ultrasonography, particularly TEE, requires formal training and expertise, and is operator dependent. Most importantly both methods are only valid in mechanically ventilated patients who are deeply sedated or paralyzed, are receiving a tidal volume of ≥8 mL/kg PBW, and are in normal sinus rhythm. The respiratory pattern of a spontaneously breathing patient is variable and inconsistent, and it is physiologically incorrect to use either method in such patients. Lastly, although the use of respiratory variation in IVC diameter to predict preload responsiveness has not been studied in patients with intraabdominal hypertension (IAH), this method will probably be inaccurate in such patients and should not be used in them. Continuous monitoring is not feasible with either of these methods.

## 5. Respiratory Variation in Arterial Blood Pressure Waveform-Derived Variables

During spontaneous breathing the respiratory changes in pleural pressure can cause cyclic changes in stroke volume and pulse pressure. During inspiration venous return and right ventricular (RV) preload increase but the left ventricular (LV) preload and SV and arterial PP decrease, and during expiration the opposite occurs—venous return and RV preload decrease but the LV preload and SV, and arterial PP increase. This phenomenon is known as pulsus paradoxus and is exaggerated when the two ventricles have to compete for space, for example, when there is pericardial tamponade or hyperinflation.

**Figure 2:**
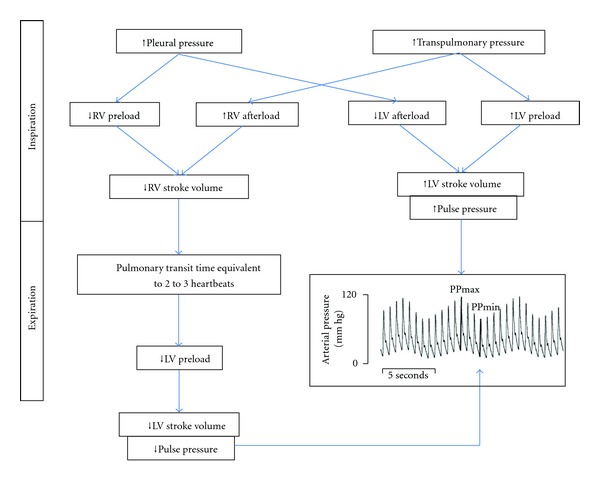
Phenomenon of reverse pulsus paradoxus. RV: right ventricular, LV: left ventricular, PP_max_: maximum pulse pressure at end-inspiration, PP_min_: minimum pulse pressure at end-expiration.

On the other hand, during mechanical ventilation the phenomenon is reversed; that is, during positive pressure inspiration venous return and RV preload decrease and RV afterload increases but the LV preload and SV, and arterial PP increase, and during expiration the opposite occurs (Figure 2). The decrease in venous return is due to the inspiratory increase in pleural pressure [17]. The increase in RV afterload is related to the inspiratory increase in transpulmonary pressure (alveolar minus pleural pressure) [18]. The decrease in RV preload and the increase in RV afterload both lead to a decrease in RV SV, which is therefore at its minimum at the end of the inspiratory period [19]. The inspiratory decrease in venous return is assumed to be the main mechanism of the inspiratory fall in RV SV [20]. On the other hand, the increase in LV preload leads to an increase in LV SV. The inspiratory decrease in RV SV leads to a decrease in LV preload after a phase lag of two-to-three heart beats because of the long transit time in the lungs [21]. Thus, the decrease in LV preload results in a decrease in LV SV, which is at its minimum during the expiratory period [19]. Two other mechanisms may also occur: positive pressure ventilation may induce a squeezing of blood out of alveolar vessels and thus transiently increase LV preload [22]; the inspiratory increase in pleural pressure may decrease LV afterload and thus facilitate LV ejection [23, 24]. This phenomenon of reverse pulsus paradoxus forms the basis for using respiratory variation in the arterial circulation to predict preload responsiveness. It will only be seen as long as the heart is still functioning on the ascending part of the Frank-Starling curve. 

Pulse pressure variation (PPV) is calculated manually after measuring the maximum PP during inspiration and minimum PP during expiration from a 30 sec printout of the arterial BP waveform. The difference between the two PP readings is divided by the average of the two PP readings and expressed as a percentage. 

Stroke volume variation (SVV) is calculated by a monitor (FloTrac Vigileo Edwards LifeSciences) that analyzes the arterial blood pressure waveform and uses a proprietary algorithm to convert PP in mmHg to SV in mL/heartbeat.

Numerous studies have shown that respiratory variation in either pulse pressure or stroke volume predicts preload responsiveness accurately. Marik et al. [7] performed a systematic review of 29 studies that included 685 patients and concluded that the mean threshold value, sensitivity, and specificity for PPV were 12.5 ± 1.6%, 89%, and 88%, respectively, and the mean threshold value, sensitivity, and specificity for SVV were 11.6 ± 1.9%, 82%, and 86%, respectively.

Although both PPV and SVV predict preload responsiveness accurately, both are only valid in mechanically ventilated patients who are deeply sedated or paralyzed, receiving a tidal volume of ≥8 mL/kg PBW and in normal sinus rhythm because small tidal volumes and spontaneous breathing make the respiratory variation too small or unpredictable, and in the case of cardiac arrhythmia PPV and SVV are the result of altered ventricular filling, not respiratory variation [25]. Moreover, recent studies have revealed additional limitations: low heart rate/respiratory rate ratio (<3.6) [26], pulmonary hypertension and right ventricular systolic dysfunction [27–29], and norepinephrine [30]. This limits their usefulness in the general medical ICU patient population.

## 6. Respiratory Variation in Pulse Oximeter Plethysmographic Waveform-Derived Variables

The pulse oximeter plethysmographic (POP) waveform resembles the peripheral arterial waveform, and respiratory variation in the amplitude of the POP waveform can be used to predict preload responsiveness. It does not require an arterial line and therefore, is a noninvasive alternative. 

Feissel et al. [31] used pulse oximeter plethysmography (Sonos 5500 Philips Medical Systems, Eindhoven, Netherlands) in 23 septic patients who were on mechanical ventilation, deeply sedated, receiving a tidal volume of ≥ 8 mL/kg PBW and in normal sinus rhythm, and showed the respiratory variation in the amplitude of the POP waveform; that is, ΔPplet of >14% was as accurate as a PPV of ≥13% in predicting preload responsiveness—defined as an increase in CI of ≥15% measured by TTE. However, calculation of the respiratory variation in the amplitude of the pulse oximeter plethysmographic waveform required sophisticated analysis on a computer.

Recently the development of plethysmographic variability index (PVI Massimo Corp.) has overcome the problem and allowed respiratory variation in the amplitude of the pulse oximeter plethysmographic waveform to be measured easily at the bedside or monitored continuously. PVI is a proprietary algorithm that allows for noninvasive, automated, continuous calculations of respiratory variation in pulse oximeter plethysmographic waveform using a pulse oximeter in mechanically ventilated patients. PVI is a measure of the dynamic change in perfusion index (PI)—the ratio of nonpulsatile to pulsatile blood flow through the peripheral capillary bed—occurring during a complete respiratory cycle [32].

Feissel et al. [33] used PVI in deeply sedated mechanically ventilated septic patients in normal sinus rhythm and showed that a value of >20 identified preload responsive patients with a sensitivity of 84% and specificity of 90%. 

Loupec et al. [34] also showed that PVI can be used to predict preload responsiveness in deeply sedated mechanically ventilated surgical ICU patients in normal sinus rhythm. 

Although PVI appears to be as accurate as PPV and SVV in predicting preload responsiveness, it too is only valid in mechanically ventilated patients who are deeply sedated or paralyzed, receiving a tidal volume of ≥8 mL/kg PBW, and in normal sinus rhythm. This limits its usefulness in the general medical ICU patient population. Moreover, it is not reliable if the peripheral perfusion is severely compromised. 

## 7. Respiratory Variation in Aortic Blood Flow Velocity

Since Doppler US allows beat-to-beat measurement of blood velocity and blood velocity is proportional to LV stroke volume, the respiratory variation in peak aortic blood flow velocity, that is, Δ*V*peak, can also be used to predict preload responsiveness.

Feissel et al. [35] used TEE in 19 ventilated patients with septic shock and normal LV systolic function and showed that a Δ*V*peak of >12% predicted preload responsiveness with a sensitivity of 100% and specificity of 89%. Δ*V*peak was calculated as the difference between maximum *V*peak and minimum *V*peak divided by the mean of the two values and expressed as a percentage.

Although an esophageal Doppler can be used instead of a TEE and can be left in place, it is also less reliable because the probe is inserted blindly, and the resulting waveform is highly dependent on correct positioning.

Limitations of both methods are similar to the ones that apply to PPV and SVV. Additional limitations precluding more widespread use are long learning curve with a lack of reproducibility, inability to obtain continuous reliable measurements, requirement for 24-hour availability, and practical problems related to the presence of the probe in the patient's esophagus [36]. 

## 8. Respiratory Variation in Brachial Artery Blood Flow Velocity

Brennan et al. [37] trained internal medicine residents to use a hand-carried US device (SonoSite Titan; Bothell, WA) with a 5 MHz broadband linear array transducer weighing 7.7 lb in 30 deeply sedated patients mechanically ventilated with tidal volume of ≥8 mL/kg PBW to measure blood flow velocity in the brachial artery and showed that Δ*V*peak-BA of >16% predicted radial arterial PPV of ≥13% with a sensitivity of 91% and specificity of 95%. Δ*V*peak-BA was calculated as the difference between maximum *V*peak and minimum *V*peak divided by the mean of the two values and expressed as a percentage.

The hand-carried US Doppler assessment of the Δ*V*peak-BA is a rapid, noninvasive bedside correlate to PPV but suffers from the same limitations; that is, it is only valid in mechanically ventilated patients who are deeply sedated or paralyzed, receiving a tidal volume of at least 8 ml/kg PBW and in normal sinus rhythm.

## 9. Electrical Impedance Tomography

This recently developed technology is another noninvasive method of measuring SVV. Electrical impedance tomography (EIT) measures changes in bioimpedance at skin electrodes to reconstruct sequences of cross-sectional functional images [38]. However, at the surface of the chest, 90% of the signal amplitude is due to breathing, and, thus, it becomes challenging to exploit the small respiratory variations in SV, which represent only 1% to 2% of the total signal strength. Conventional EIT postprocessing techniques are unable to analyze such low-amplitude events. Therefore, Maisch et al. [39] developed a novel method to determine SVV in the descending aorta by analyzing sequences of EIT images in the frequency domain (SVV_EIT_) and tested it in an animal study. A wide range of hemodynamic conditions were induced in 8 pigs by mechanical ventilation at different levels of positive endexpiratory pressure (0–15 cm H_2_O) and with tidal volumes of 8 and 16 mL/kg of body weight and by hypovolemia due to blood withdrawal with subsequent retransfusion followed by infusions of hydroxyethyl starch. Aortic SVV measured by EIT and compared to SVV derived from an aortic ultrasonic flow probe and from arterial pulse contour analysis showed significant correlation (*r* ^2^ = 0.69; *P* < 0.001, and *r* ^2^ = 0.73; *P* < 0.001, resp.) [39]. EIT appears to be a promising new noninvasive method of determining preload responsiveness; however, it has not been studied in humans yet.

## 10. Passive Leg Raising Maneuver

Raising the legs to 45 degrees from the supine or semirecumbent position mobilizes the reservoir of blood in the legs and the splanchnic circulation towards the chest and results in an endogenous volume challenge. If the heart is preload responsive, that is, it is functioning on the ascending portion of the Frank-Starling curve, PLR maneuver will result in an increase in CI within one minute. On the other hand, if the heart is not preload responsive, that is, it is functioning on the plateau part of the Frank-Starling curve, PLR will not result in an increase in CI [40].

The hemodynamic effect of PLR is similar to the intravenous infusion of fluids [41] and is not affected by the presence of spontaneous breathing. Moreover, since the mean change in CO after PLR is measured over several heartbeats, it is not affected by cardiac arrhythmias. Lastly, the hemodynamic effect is reversible.

A recent meta-analysis [10], which pooled the results of nine studies and included a total of 353 patients, confirmed the accuracy of the PLR maneuver in predicting preload responsiveness with a pooled sensitivity and specificity of 89.4% (84.1–93.4%) and 91.4% (85.9–95.2%), respectively. The pooled area under the receiver operating characteristics curve (AUC) was 0.95 (0.92–0.97) (Table 1).

Although Jabot et al. [42] found significant difference in hemodynamic response to PLR performed by starting from supine versus semirecumbent position, the meta-analysis (10) did not show any significant difference.

Moreover, the PLR maneuver cannot accurately predict preload responsiveness in patients with intra-abdominal hypertension (IAH) because venous return is impaired in such patients [43]. 

Most importantly, although the PLR maneuver is comparable in accuracy to PPV and SVV in predicting preload responsiveness (Table 1) and has the advantage of not being affected by the mode of breathing, tidal volume, or cardiac rhythm and therefore can be used in patients who are breathing spontaneously or have arrhythmias, and unlike an actual fluid challenge has no adverse effects, it requires measurement of SV (or CO) or alternatively a surrogate, for example, arterial blood flow velocity or PP.

Different methods of SV (or CO) measurement have been validated with the PLR maneuver, that is, PAC with continuous CO (cCO) monitoring capability using thermodilution technique [44], PiCCO thermodilution technique [45], FloTrac Vigileo [46], TTE [47, 48], transthoracic Doppler US (USCOM, Sydney, Australia) [49], or NICOM [50, 51]. Measurement of arterial blood flow velocity as a surrogate for SV (or CO) has also been validated with the PLR maneuver, that is, aortic blood flow velocity measured by esophageal Doppler US [52, 53] or femoral artery blood flow velocity measured by Doppler US [54] (Table 2).

The meta-analysis [10] also showed that the accuracy of the PLR maneuver is independent of the method used to measure the SV (or CO) or arterial blood flow velocity—PAC with cCO, PiCCO, FloTrac Vigileo, echocardiography, Doppler US. Of note, however, the studies using NICOM were not included in the meta-analysis because they had not been published at the time of the meta-analysis.

On the other hand when PP is used as a surrogate for SV (or CO) the accuracy of the PLR maneuver is lower: pooled sensitivity of 59.5% (47.4–70.7%), specificity of 86.2% (75.3–93.5%), and AUC of 0.76 (0.67–0.86). This is because PP is not a direct measure of SV and is affected by the compliance of the vessel [10].

PAC with cCO monitoring capability using thermodilution technique, PiCCO, and FloTrac Vigileo are invasive and require a pulmonary artery catheter, both an internal jugular (IJ) or subclavian (SC) central venous line (CVL) and a femoral arterial line, and an arterial line, respectively, and, therefore, may not be feasible in the emergency room or on the floor during the initial resuscitation of sepsis. Moreover, unlike FloTrac Vigileo, PiCCO requires frequent recalibration. On the other hand, US techniques are non- or semi-invasive but require formal training and are operator dependent, and some like TTE or TEE are not continuous. Although an esophageal Doppler can be used instead of a TEE and can be left in place, it is also less reliable because the probe is inserted blindly, and the resulting waveform is highly dependent on correct positioning. Additional limitations precluding more widespread use are long learning curve with a lack of reproducibility, inability to obtain continuous reliable measurements, requirement for 24 hour availability, and practical problems related to the presence of the probe in the patient's esophagus [36].

USCOM and NICOM are two promising new technologies that measure CO non-invasively.

USCOM (Uscom Ltd., Sydney, Australia) is a transthoracic ultrasonic CO monitor that uses continuous wave Doppler technique to measure CO and has been validated with the PLR maneuver in critically ill patients. Thiel et al. [49] studied 89 medical ICU patients requiring volume expansion and found that a PLR-induced increase in SV ≥15% predicted preload responsiveness with a sensitivity of 81%, specificity of 93%, negative predictive value of 85%, and positive predictive value of 91%. 

NICOM (Cheetah Medical, Washington, WA, USA) is a noninvasive CO monitor that is based on bioreactance technique and is comparable in accuracy to the invasive techniques of thermodilution (PAC with cCO, PiCCO) and arterial BP waveform analysis (FloTrac Vigileo) [55–59]. It consists of a 75 kHz sine wave generator and four dual electrode stickers that are used to establish electrical contact with the body. Measurement of CO is based on analysis of relative phase shifts of an oscillating current that occurs when this current traverses the thoracic cavity [60]. 

NICOM has been validated with the PLR maneuver in critically ill patients and shown acceptable accuracy [50, 51]. Lamia et al. [50] studied 11 hemodynamically unstable patients with spontaneously breathing activity in a respiratory critical care unit and found that a PLR-induced increase in SV of ≥9% predicted an increase in SV of ≥15% after a 500 mL NS bolus with a sensitivity of 100% and a specificity of 80%. Benomar et al. [51] studied 75 postcardiac surgery patients and found that PLR-induced increase in CO of ≥9% predicted an increase in CO of ≥9% after a 500 mL colloid bolus with a sensitivity of 68% and specificity of 95%. 

Lakhal et al. [61] showed that combining the PLR maneuver with CVP can improve its accuracy since a ≥2 mmHg increase in CVP in a nonresponsive patient indicates that PLR guaranteed an adequate endogenous volume challenge. 

## 11. Actual Fluid Challenge 

Several decades ago, Weil and Henning [62] proposed the fluid challenge technique, based on the “2–5 rule” using the CVP and the “3–7 rule” for the PAWP. CVP was measured at 10 min intervals. If the change in CVP was <2 mmHg, the infusion was continued. If it was in the 2–5 mmHg range, the infusion was held and CVP remeasured after 10 min. If the change was an increase of >5 mmHg, the infusion was stopped. PAWP was used in a similar manner but with different cut-offs, that is, <3 mmHg, 3–7 mmHg, and >7 mmHg. 

Recently Vincent and Weill [63] proposed a modified fluid challenge technique that incorporates 4 decisions phases: type of fluid, rate of administration, clinical end-points and pressure safety limits. Clinical end-points are usually correction of the hemodynamic abnormality that prompted the need for fluid, that is, hypotension, tachycardia, or oliguria. Although this technique like the one mentioned previously (“2–5” rule and “3–7” rule) has not been validated either, it appears to offer several advantages: quantitative goals together with limits are imposed, fluid deficits are more rapidly corrected, and fears of large volumes are minimized. Moreover, the protocol identifies cardiac failure early, based on early increases in filling pressures to threshold levels, and directs the clinician to search for causes of perfusion failure other than hypovolemia. See Table 3 for two illustrative examples of the technique. 

It is important to realize that a fluid challenge technique that uses SV (or CO) or its surrogate as the end-point is the most accurate way of performing a fluid challenge maneuver and has been used as the gold standard for comparison in studies of the PLR maneuver to predict preload responsiveness. An increase in SV (or CO) ≥ of 15% defines preload responsiveness [10]. However, the requirement for measurement of SV (or CO) or alternatively a surrogate, for example, arterial blood flow velocity or pulse pressure, makes the fluid challenge maneuver that uses SV (or CO) or its surrogate as the end-point a more complex maneuver than the usual fluid challenge that uses other albeit less accurate end-points that is, BP, HR, and urine output.

An actual fluid challenge maneuver may not be appropriate in some clinical situations where an intravenous fluid bolus may prove harmful, for example, severe acute respiratory distress syndrome (ARDS) or anuric acute tubular necrosis (ATN).

## 12. Summary of the Approach to Optimizing Preload

**Figure 3:**
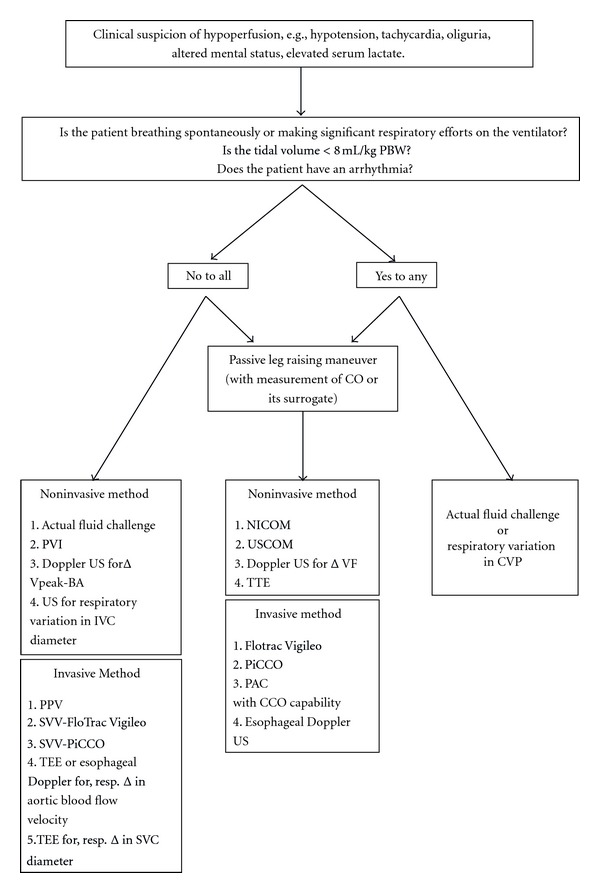
Approach to optimizing preload. PBW: predicted body weight, CO: cardiac output, PVI: pleth variability index, Δ*V*peak-BA: respiratory variation in peak brachial arterial blood flow velocity, US: ultrasonography, IVC: inferior vena cava, PPV: pulse pressure variation, SVV: stroke volume variation, TEE: transesophageal echocardiography, SVC: superior vena cava, NICOM: noninvasive cardiac output monitor, USCOM: ultrasonic cardiac output monitor, ΔVF: change in femoral artery blood flow velocity, CCO: continuous cardiac output, TTE: transthoracic echocardiography, PAC: pulmonary artery catheter, CVP: central venous pressure.

The choice of the method used to determine preload responsiveness depends upon patient-related factors as well as the available technology and expertise. The most important patient-related factors are mode of breathing (spontaneous versus deeply sedated or paralyzed on mechanical ventilation), tidal volume (< or ≥8 ml/kg PBW), and cardiac rhythm (normal sinus rhythm versus arrhythmia) (Figure 3, Table 4). Other factors that need to be considered are risks associated with the method, for example, PAC with cCO monitoring capability using thermodilution technique; PiCCO and FloTrac Vigileo which require a pulmonary artery catheter, both an IJ or SC CVL and a femoral arterial line, and an arterial line, respectively, are invasive and associated with risks of bleeding and infection which become even more important in critically ill septic patients particularly those with coagulopathy or neutropenia (Table 5). Noninvasive methods, therefore, present a very appealing alternative. Moreover, non-invasive methods like PVI and NICOM are faster compared to placing a CVL or arterial line which can be time consuming. Last but not least is the cost of technology. Although it appears that US may be an expensive tool to determine preload responsiveness, it is important to keep in mind that a US machine being reusable pays for itself in the long run. Similarly, other technologies that seem expensive are also fairly cost-effective when it is realized that the monitor (e.g., FloTrac Vigileo, PVI, NICOM) or device which is the main expense is a onetime investment and any disposable accessories (e.g., FloTrac sensors, finger sensors for PVI, NICOM electrode cables) are cheap (Table 6).

A PLR maneuver or actual fluid challenge combined with measurement of SV (or CO) or its surrogate is the best method to accurately predict preload responsiveness regardless of the mode of breathing, tidal volume, and cardiac rhythm. 

If the patient is deeply sedated or paralyzed on a ventilator, is receiving a tidal volume of ≥8 mL/kg PBW, and is in normal sinus rhythm, “dynamic” parameters based on respiratory variation in the venous or arterial circulation, that is, IVC or SVC diameter, PPV, SVV, ΔPleth, PVI, aortic or brachial artery blood flow velocity, can accurately predict preload responsiveness. However, such a situation is commonly seen only in patients under general anesthesia in the operating room setting, and in fact most of the original studies using PPV and SVV were performed in such a setting. 

On the other hand, most of the ICU patients are breathing spontaneously or making significant respiratory efforts on the ventilator. Under such circumstances, there are four options:

1. PLR maneuver or actual fluid challenge maneuver combined with measurement of SV (or CO) or its surrogate,
2. use of respiratory variation in CVP,
3. the fluid challenge technique proposed by Vincent and Weil [63].

Both PLR maneuver and actual fluid challenge maneuver combined with measurement of SV (or CO) or its surrogate require continuous real-time measurement of SV (or CO), that is, PAC with cCO, PiCCO, FloTrac Vigileo, USCOM or, NICOM, or alternatively, its surrogate, that is, aortic blood flow velocity measured by esophageal Doppler US or femoral artery blood flow velocity measured by Doppler US. Moreover, a PLR maneuver cannot be used in patients with IAH or pelvic fractures.

Use of respiratory variation in CVP to predict preload responsiveness requires that the inspiratory effort be significant enough to cause a 2 mmHg drop in PAWP, and therefore in the absence of a PAC to confirm such a significant respiratory effort the technique becomes subjective and dependent on observing the patient. Moreover, in a patient who is using expiratory abdominal muscles the release of abdominal muscle contraction may be confused for an inspiratory fall in CVP.

An actual fluid challenge maneuver may not be appropriate in some clinical situations where an intravenous fluid bolus may prove harmful, for example, severe ARDS or anuric ATN.

(4) Lastly, it is important to keep in mind that the “dynamic” parameters based on respiratory variation in the venous or arterial circulation, that is, IVC or SVC diameter, PPV, SVV, ΔPleth, PVI, aortic or brachial artery blood flow velocity, can still be used to predict preload responsiveness in a ventilated patient *if* the ventilated patient is *temporarily* paralyzed and tidal volume is *temporarily* increased for a couple of minutes to 8–10 mL/kg PBW (unless contraindicated). Although they still cannot be used when the cardiac rhythm is irregular, the recent development of a new algorithm (SVVxtra Edwards Lifesciences, Irvine, CA, USA) *might* allow SVV to be used even *if *the cardiac rhythm is irregular. The new SVV algorithm is designed to restore the respiratory component of the arterial blood pressure waveform despite multiple ectopic heart beats. In a recent animal study Canneson et al. [64] used this new algorithm in 8 anesthetized and mechanically ventilated pigs. Multiple extrasystoles were induced by right ventricular pacing (25% of heart beats). Arterial blood pressure waveforms were recorded, and SVV was computed from the new and from the standard algorithm. A positive response to a bolus of 7 mL/kg of 6% hydroxy ethyl starch was defined as >15% increase in CO. The new SVV was higher in responders than in nonresponders (19 ± 5% versus 12 ± 3%, *P* < 0.05), whereas the standard SVV was similar in the two groups (29 ± 8% versus 26 ± 11%, *P* = 0.4). Receiver operating characteristic curve analysis showed that the new SVV was an accurate predictor of preload responsiveness (sensitivity = 86%, specificity = 85%, best  cutoff  value = 14%, AUC = 0.892 ± 0.052), whereas the standard SVV was not (AUC = 0.596 ± 0.077) [64].

## Figures and Tables

**Table 1:** Accuracy of various parameters used to predict preload responsiveness [7, 10].

Parameter	Technology	AUC with 95% CI
PLR^∗^	Various methods of CO measurement	0.95 (0.92–0.97)
PPV	Arterial BP waveform	0.94 (0.93–0.95)
SVV	Arterial BP waveform analysis by proprietary algorithm	0.84 (0.78–0.88)
LVEDAI	Echocardiography	0.64 (0.53–0.74)
GEDV	Thermodilution	0.56 (0.37–0.67)
CVP	Central venous catheter	0.55 (0.48–0.62)

PLR: passive leg raising, PPV: pulse pressure variation, SVV: stroke volume variation, LVEDAI: left ventricular end-diastolic area index, GEDV: global end-diastolic volume, CVP: central venous pressure, AUC: area under receiver operating characteristics curve.

**Table 2:** Different methods of measuring CO or arterial blood flow velocity^∗^ during PLR maneuver.

Invasive	Semi-invasive	Noninvasive
*Thermodilution*	*US*	*US*
PAC (transpulmonary thermodilution)	^ ∗^Esophageal Doppler	Transthoracic echocardiography
PiCCO (aortic transpulmonary thermodilution)		Transthoracic USCOM
*Arterial BP waveform analysis*		^ ∗^Femoral arterial Doppler
FloTrac Vigileo		*Bioreactance*
		NICOM

CO: cardiac output, PLR: passive leg raising, PAC: pulmonary artery catheter, *US*: ultrasound, USCOM: ultrasonic cardiac output monitor, NICOM: noninvasive cardiac output monitor.

**Table 3:** Critical components of the fluid challenge and one example of their application in a hypothetical patient (MAP of 65 mmHg and a CVP of 12 mmHg; two possible types of response are presented) [63].

Example	example 1			example 2		
(1) Type of fluid: Ringer's lactate	Baseline	+10 mins	+20 mins	Baseline	+10 mins	+20 mins
(2) Rate of infusion: 500 mL/30 mins						
(3) Clinical end-points: MAP of 75 mmHg	MAP 65	MAP 70	*MAP 75*	MAP 65	MAP 67	MAP 60
(4) Pressure safety limits: CVP of 15 mmHg	CVP 12	CVP 13 Continue	CVP 14 Stop	CVP 12	CVP 14 Continue	*CVP 15* Stop
	*Successful fluid challenge*			*Unsuccessful fluid challenge*		

MAP: mean arterial pressure, CVP: central venous pressure, mins: minutes [63].

**Table 4:** Advantages and disadvantages of the various dynamic parameters used to predict preload responsiveness.

Method	Advantages	Disadvantages
Respiratory changes in CVP	Most critically ill septic patients have an IJ or SC CVL	It requires that the inspiratory effort be significant—a fall in PAWP of ≥2 mmHg was used in the original study by Magder et al. [11]
	It can be used in spontaneously breathing patients	

Respiratory changes in IVC diameter	It is non-invasive and requires an ultrasound with M-mode which is now becoming widely available	It is only reliable in mechanically ventilated patients who are receiving ≥8 mL/kg PBW tidal volume, are not making any significant respiratory efforts, and are in NSR
	It is easy to learn and teach	It may not be reliable in conditions associated with IAH, for example, obesity, massive ascites, abdominal compartment syndrome
	It can be easily repeated as often as necessary	

Respiratory changes in SVC diameter	It is more accurate than respiratory change in IVC diameter	It is semi-invasive and requires TEE and expertise in using it
		It is not continuous
		It too is only reliable in mechanically ventilated patients who are receiving ≥8 mL/kg PBW tidal volume, are not making any significant respiratory efforts, and are in NSR

PPV	PPV can be calculated manually from a 30 sec printout of the arterial blood pressure waveform	It is invasive and requires an arterial line
		It is only reliable in mechanically ventilated patients who are receiving ≥8 mL/kg PBW tidal volume, are not making any significant respiratory efforts, and are in NSR

SVV-FloTrac Vigileo	It does not require frequent recalibration	It is invasive and requires an arterial line
	It provides additional data: SV, CO	It is only reliable in mechanically ventilated patients who are receiving ≥8 mL/kg PBW tidal volume, are not making any significant respiratory efforts, and are in NSR

SVV-PiCCO Plus	It provides additional data: SV, CO, TBV, and EVLW	It is invasive and requires an IJ or SC CVL and a femoral arterial line with a thermistor
		It requires frequent recalibration
		It is only reliable in mechanically ventilated patients who are receiving ≥8 mL/kg PBW tidal volume, are not making any significant respiratory efforts, and are in NSR

PVI	It is noninvasive	It is only reliable in mechanically ventilated patients who are receiving ≥8 mL/kg PBW tidal volume, are not making any significant respiratory efforts, and are in NSR
	It is easy to use	It is not reliable if peripheral perfusion is severely compromised
	It does not require calibration	

		Semi-invasive and requires TEE or esophageal Doppler US and expertise in using it
		It is only reliable in mechanically ventilated patients who are receiving ≥8 mL/kg PBW tidal volume, are not making any significant respiratory efforts, and are in NSR
		It suffers from additional limitations:
Respiratory changes in aortic blood flow velocity	Esophageal Doppler US monitoring uses a smaller esophageal probe than TEE and therefore is less invasive; it can also be left in place for continuous monitoring; it also requires less training to use and is less expensive	Long learning curve with a lack of reproducibility
		Inability to obtain continuous reliable measurements
		Requirement for 24-hour availability
		Practical problems related to the presence of the probe in the patient's esophagus
		As esophageal Doppler probes are inserted blindly, the resulting waveform is highly dependent on correct positioning

Respiratory changes in brachial artery blood flow velocity	It is non-invasive and requires only a US with Doppler which is now becoming widely available in ICUs	It is only reliable in mechanically ventilated patients who are receiving ≥8 mL/kg PBW tidal volume, are not making any significant respiratory efforts, and are in NSR
	It is easy to learn and teach as demonstrated by a study where residents used it after learning the technique	

PLR maneuver	It can be used in spontaneously breathing patients	It requires continuous CO monitoring by a technology with a rapid response time, for example, USCOM, NICOM, FloTrac Vigileo, PiCCO, or PAC with such capability
	It can be used in patients with arrhythmias	
	It can be completely noninvasive if CO is measured by a noninvasive method, for example, USCOM or NICOM	

CVP: central venous pressure, IJ: internal jugular, SC: subclavian, CVL: central venous line, PAWP: pulmonary artery wedge pressure, IVC: inferior vena cava, PBW: predicted body weight, NSR: normal sinus rhythm, IAH: intra-abdominal hypertension, SVC: superior vena cava, TEE: transesophageal echocardiography, PPV: pulse pressure variation, SVV: stroke volume variation, SV: stroke volume, CO: cardiac output, TBV: thoracic blood volume, EVLW: extravascular lung water, US: ultrasound, USCOM: ultrasonic cardiac output monitor, NICOM: noninvasive cardiac output monitor.

**Table 5:** Complications of vascular catheters.

Immediate	Delayed
Central venous catheter and pulmonary artery catheters	

Bleeding	Infection
Retroperitoneal hematoma (with femoral approach)	Venous thrombosis, pulmonary emboli
Arterial puncture	Catheter migration
Arrhythmia	Catheter embolization
Air embolism	Myocardial perforation
Thoracic duct injury (with left SC or left IJ approach)	Nerve injury
Catheter malposition	
Pneumothorax or hemothorax	

Arterial catheters	

Bleeding	Infection
Retroperitoneal hematoma (with femoral approach)	Thrombosis
	Limb ischemia
	Cerebral embolization
	Nerve injury
	Pseudoaneurysm
	Arteriovenous fistula

IJ: internal jugular, SC: subclavian.

**Table 6:** Comparison of the cost of various technologies^∗^.

	Cost of the equipment	Cost of the consumables
Flotrac Vigileo	EV1000 Clinical	FloTrac sensors: *£*85–130 dependent upon volume/commitment
	Platform: Placed (*£*0) to *£*14,450	
	Vigileo Monitor:	
	Placed (*£*0) to *£*6,985	

PVI	*£*1995	Finger sensor costs *£*8 per patient

Esophageal Doppler (CARDIOQ-ODM) for 6, 12, 24, 72, 240 hour use	*£*12,000	A range of probes is available ranging from *£*73–*£*96.
		Additionally, longer duration probes are available ranging from *£*116–*£*128

USCOM	*£*16,000	No consumables required

NICOM	*£*4995	Disposable patient sensors. Cost varies depending on quantity—if 200 bought, then cost is *£*40 per patient

^∗^Information in this table obtained from the UK NHS Technology Adoption Centre's adoption pack 2012. http://www.ntac.nhs.uk/web/FILES/IOFM_Adoption_pack_final_080512.pdf.
